# Ionizing radiation effects on osseointegration: a pre-clinical study

**DOI:** 10.1590/1807-3107bor-2024.vol38.0112

**Published:** 2024-12-09

**Authors:** Roberta de Oliveira Alves, Guilheme José Pimentel Lopes de Oliveira, Rita Catarina de Oliveira, Pedro Henrique Justino Oliveira Limirio, Marcela Claudino, Darceny Zanetta-Barbosa, Priscilla Ferreira Barbosa Soares

**Affiliations:** (a) Universidade Federal de Uberlândia – UFU, School of Dentistry, Department of Periodontology and Implantology, Uberlândia, MG, Brazil.; (b) Universidade Estadual de Ponta Grossa – UEPG, Department of Dentistry, Ponta Grossa, PR, Brazil; (c) Universidade Federal de Uberlândia – UFU, School of Dentistry, Department of Oral and Maxillofacial Surgery, Uberlândia, MG, Brazil.

**Keywords:** Radiation, Ionizing, Osseointegration, Bone and Bones

## Abstract

The purpose of this study was to evaluate the effect of a single dose of ionizing radiation (30 Gy) on the osseointegration of implants in the rabbit tibia. Twenty rabbits received two dental Morse-tapered junction implants and one implant in each tibia. The animals were randomly divided into two groups (n=10), non-irradiated (NoIr) and irradiated (Ir), wherein the Ir group received a single dose of 30 Gy radiation 2 weeks after implant installation. Microtomographic analyses (BV/TV) and histomorphometric assessments (BIC and BABT) were performed 4 weeks after implant installation. One-way ANOVA, Tukey's test, and Student's t-test (α=0.05) were used for data analysis. The results showed that BV/TV did not differ significantly between the Ir and NoIr groups (P = 0.071). In the histomorphometric analysis, neither BIC nor BABT showed significant differences between the NoIr and Ir groups (p>0.05). In conclusion, ionizing radiation in dental implants does not appear to interfere with osseointegration when installed prior to irradiation.

## Introduction

Radiotherapy is a widely used treatment for tumors in the head and neck region; however, despite being effective for several types of cancer and even with advances in the medical field regarding treatment planning and the form of application of radiotherapy, some patients still suffer from adverse effects related to this therapy.^1^ The occurrence of these side effects is related to the fact that radiation affects not only the tumor cells, but also healthy cells adjacent to the tumor.^2^ Tissue damage depends on factors such as tissue sensitivity to radiation, cell organization, and radiation dose.^2,3^

Bone tissue suffers from the deleterious effects of ionizing radiation, as it has the potential to absorb more radiation than other tissues.^4,5^ Previous studies have shown that irradiation causes important changes in bone cell metabolism, which have negative influences on bone architecture, mechanical properties, and remodeling.^6–8^ Tooth extraction with a doubtful prognosis is highly recommended within the treatment plan of patients undergoing radiotherapy due to the impossibility of extracting these teeth during the active phase and after radiotherapy sessions.^9,10^ Thus, these patients may experience events that significantly impair their quality of life since radiation can impair even the oral rehabilitation of these patients.^11–13^

Implant rehabilitation has been increasingly used in clinical practice to treat all types of edentulism with high success rates.^14,15^ However, some risk factors may interfere with the success of dental implant therapy, and patients who undergo radiation therapy may have reduced success in the osseointegration process compared to the general population.^13,16^ Various methods are employed to evaluate osseointegration, including microtomographic analysis^7,17^, radiographs,^18,19^ histomorphometry,^17,19,20^ biomechanical tests,^7^ push-out/pull-out testing, and clinical evaluation.^19^ There are some questions raised in the literature whether implants previously installed at some point in life prior to radiotherapy treatment would also be at risk of being affected, such as in implants that are installed after radiotherapy treatment.

Owing to these conflicting results, further investigations are required to gather more information regarding the impact of radiotherapy on the osseointegration of implants. In this study, we aimed to assess the effects of ionizing radiation on osseointegrated implants in rabbit tibiae.

## Material and methods

The pre-clinical in vivo study strictly adhered to the ARRIVE guidelines and encompassed all relevant facets. Ethical clearance for our animal experimentation procedures was obtained from the Bioethics Committee for Animal Experimentation (CEUA # 093/12) at the Federal University of Uberlândia. Furthermore, our research conformed to the regulatory directives established by the National Council for Animal Control and Experimentation (CONCEA), which operates under the auspices of the Ministry of Science, Technology, and Innovation (MCTI) in Brazil, as outlined in Law 11.794, dated 08/19/2008.

Twenty New Zealand white male rabbits that weighed between 3.0 and 3.5 kg were included in the study. A 2-week acclimatization period was provided for all animals prior to the commencement of any experimental procedures. During this period, each rabbit was individually housed in a standardized cage equipped with appropriate bedding and nesting materials. Environmental conditions were maintained at a constant temperature of 20°C, with controlled humidity, and adhered to a 12-hour circadian rhythm. The rabbits were fed a diet consisting of standard laboratory pellets, and water was provided ad libitum. Individuals responsible for animal care were kept unaware of the group assignments.

Each rabbit received a single implant in the tibia. Following this procedure, the animals were randomly allocated into one of two groups (n = 10 each): the non-irradiated group (NoIr), in which the animals were not subjected to ionizing radiation exposure, and the irradiated group (Ir), in which external irradiation was administered to both tibias 2 weeks after the implantation surgery. Following irradiation, the bone surrounding the implants was analyzed to assess the bone microarchitecture and morphological properties.

### Surgical procedure

The animals were subjected to fasting before the surgical procedure. To maintain sterility at the surgical site, the fur on the animals’ legs was shaved, and the tibial regions were cleaned using a 0.2% solution of chlorhexidine (Rioquimica, São José do Rio Preto, SP, Brazil). Anesthesia was administered to the animals through intramuscular injection, involving a combination of 0.25 mg of ketamine per kilogram of body weight (Ketamina Agener^®^, produced by Agener União Ltda., São Paulo, SP, Brazil) and 0.5 mg of xylazine per kilogram of body weight (Rompum^®^ by Bayer S.A., São Paulo, SP, Brazil). To minimize stimulation during the surgery and promote vasoconstriction, the anesthesia was accompanied by the use of 2% lidocaine and 1:100,000 epinephrine (Alphacaine^®^ at 0.5 - 1 ml per site, manufactured by DFL, Rio de Janeiro, RJ, Brazil). Incisions measuring 3 cm were made in both tibias. The soft tissue and periosteum were removed and precise subperiosteal dissection was performed to expose the proximal tibia. In the diaphysis region, primarily consisting of cortical bone, Grade 4 titanium dental implants with a Morse taper junction, measuring 3.75 mm in diameter and 7.0 mm in length (Titamax Acqua CM, Neodent^®^, Curitiba, PR, Brazil), were inserted into each animal. One implant was placed in the left tibia and the other in the right tibia. The implantation process followed a gradual sequence of drills, with continuous irrigation using a 0.9% sodium saline solution, in accordance with the manufacturer's instructions. All drilling procedures were performed at 1200 rpm, with the depth parameter determined solely by the penetration of the outer cortical bone. The soft tissues were sutured in distinct layers using an interrupted suture technique (#5.0 nylon sutures by Ethicon^®^, Johnson & Johnson Medical Ltd., Blue Ash, Ohio, United States). To prevent infection, daily intramuscular injections of cefazolin (250 mg/kg; Ourofino, São Paulo, SP, Brazil) was administered for a duration of 1 week. To manage pain, a dose of 0.3 mg/kg of the anti-inflammatory Meloxicam^®^ (Ourofino) was provided. Each rabbit was housed individually at room temperature and provided with adequate food and water. Daily observations were conducted during the postoperative phase to monitor behavioral changes indicative of distress and weight loss. Two weeks post-surgery, the animals were randomly allocated into two groups: one that did not receive irradiation and the other that received irradiation.

### Irradiation protocol

Two weeks after the implant placement, irradiation was performed in the irradiated group. During the irradiation sessions, animals in the irradiated group were maintained under sedation by intramuscular injection of a combination of 1.3 ml ketamine (100 mg/kg) and xylazine chlorate (7 mg/kg body weight). The hind legs of each rabbit were irradiated with a single dose of 30 Gy. A 5-mm bolus was administered to ensure full build-up. The tibial metaphysis region of the hind leg was designated as the irradiation zone. A single dose of radiation was delivered with a source–skin distance of 60 cm and a field measuring 15 × 15 cm with a direct electron beam of 6 MeV (Varian 600-C^®^ Varian Medical Systems Inc, Palo Alto, California, USA). The dose rate was 400 cGy/min. After irradiation, the veterinarian closely monitored the skin, hair, weight, and appetite of the rabbits for 2 weeks.

### Animal sacrifice and sample preparations

All animals were sacrificed 4 weeks after implant installation. Animals were anesthetized with 2.5% thiopental and sacrificed with an intravenous injection of 19% potassium chloride (Ariston Chemical and Pharmaceutical Industry Ltd., São Paulo, SP). The overlying soft tissues were removed, and the tibiae were fixed with 10% formaldehyde and stored in phosphate-buffered saline (PBS) solution until the samples were scanned using micro-computed tomography (micro-CT). After scanning, implants were subjected to histomorphometric analysis.

### Microcomputed tomography (micro-CT) analyses

The images underwent reconstruction, spatial repositioning, and analysis employing dedicated software (NRecon, Data Viewer, CTAnalyser, Aatselaar, Belgium). The region of interest (ROI) was meticulously defined as a 0.5-mm circular area encompassing the entire diameter of the implant. This ROI was designated as the Total Volume (0.5-mm margin around the implants, 4.5 × 3.2 mm). In instances where cover screws were not applied to some implants, bone formation occurred within the prosthetic platform. To mitigate any interference of bone formation with the analysis of mineralized tissue volume around the implant, a second ROI was established to exclude the platform volume. By comparing the results obtained from both ROIs, the volume of bone formation could be precisely determined using the formula: Total Volume - Platform Volume = Volume of mineralized tissues. The analysis employed a threshold of 25-90 shades of gray, and the volume values of mineralized tissue around the implants were expressed as percentages. All analyses were conducted by a skilled examiner who remained blinded to the experimental groups throughout the process.

### Histomorphometric procedures

Following the imaging process, the right tibiae underwent dehydration in a sequence of ethanol solutions (60%-100%). Subsequently, they were infiltrated and polymerized using a light-curable resin (Technovit 7200 VLC, Kultzer Heraus GmbH & CO, Wehrheim, Germany). Blocks that encompassed both the implant and bone tissue were precisely sectioned at the central point utilizing a wear-and-tear system (Exakt Apparatebau, Hamburg, Germany). The resulting sections were approximately 45-μm thick and subjected to staining with Stevenel's blue combined with acid fuchsin. These stained sections were then examined under an optical microscope (DIASTAR - Leica Reichert & Jung products, Wetzlar, Germany) at a 20X magnification. Histomorphometric assessment was conducted using image analysis software (ImageJ, San Rafael, CA, USA). The percentage of bone-implant contact (% BIC) and the bone area between implant turns (% BABT) were independently evaluated for the initial six implant casts. All analyses were carried out by a proficient examiner who remained blinded throughout the process.

### Statistical analysis

SigmaPlot version 13.1 (Systat Software Inc., San Jose, CA, USA) was used for the statistical analysis. The micro-CT and histomorphometric data were assessed for normal distribution using the Shapiro-Wilk test (P>0.05) and for equality of variances using the Levene's test. One-way analysis of variance (ANOVA) was conducted for micro-CT values, followed by Tukey's test for multiple comparisons. Student's t-tests were used to analyze the pull-out data. All statistical tests were performed at a significance level of 95%.

## Results

### Micro-CT analysis – bone microarchitecture

In the analysis of bone microarchitecture using MicroCT, the results concerning the relationship between the bone volume and total tissue volume (BV/TV) did not show a statistically significant difference (P = 0.071) between the Ir (32.89 ± 1.32) and the NoIr (31.14 ± 1.35) groups.

### Histomorphometric analysis

**Figure 1:**
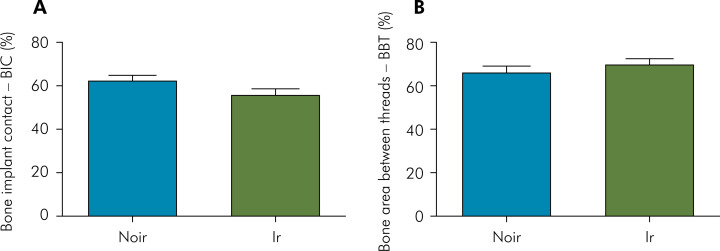
Effect of irradiation protocol regarding bone-to-implant (BIC) and bone area between threads (BABT). The data represent mean ± SE (n = 5 animals/group). No differences were detected between the groups.

The histomorphometric results are shown in Figure 1. In the NoIr group, a %BABT of 65.48 ± 8.14 and a %BIC of 61.38 ± 8.61% were observed, while in the Ir group, the corresponding values were %BABT of 70.12 ± 10.21% and %BIC of 51.77 ± 15.86%. No significant differences were found between the Ir and NoIr groups for any of the analyzed parameters (p>0.05).

### Qualitative Microscopic Analysis

**Figure 2:**
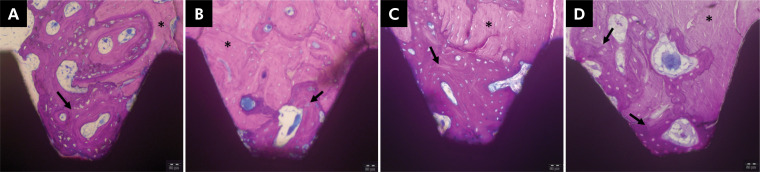
Sections of the implants and the surrounding tissue; A and B: Ir group; C and D: NoIr group. The presence of mature bone (asterisks) and newly-formed bone in the implant surface (narrow).

A qualitative microscopic study revealed the formation of new bone tissue in both groups, identifiable by a bluish color, adjacent to the implant surface in all analyzed samples. New bone matrix emerging between the implants and bone walls was observed, indicating contact osteogenesis (Figure 2, asterisk). Both implant surfaces were surrounded by newly formed bone, characterized by the trabeculae of immature bone and thickening of the cortical bone near the implants in both the irradiated and non-irradiated groups.

Mature bone tissue was present along the lamina (Figure 2, arrow) in both the groups. Osteocytes were prominent in this region. A thin layer of osteoblasts and newly formed bone, along with fibrovascular tissues, was observed at the bone-implant interface. Gradual progression from young bone to more advanced maturity was also identified in both Ir and NoIr groups, although cell counting was not performed.

## Discussion

The results of this study showed that ionizing radiation had no influence on the process of implant osseointegration in terms of the relationship between bone volume and total tissue volume, percentage of bone-implant contact, and bone area between the implant threads.

Despite the limitations of using animals to extrapolate clinical situations, they are essential to guide clinical studies aimed at validating protocols. The rabbit tibia model employed in this study is recognized as suitable for assessing the biomechanical properties related to the osseointegration process following implantation.^21^ This animal model shares notable similarities with the Haversian systems found in human individuals and stands out due to its remarkable bone turnover rate, which is approximately three times faster, enabling analyses to be conducted at short intervals during the osseointegration process.^22^ Additionally, a 4-week interval was adopted between implant placement and animal sacrifice in an effort to mimic an early stage of osseointegration akin to what occurs in human patients, providing a foundation for current therapeutic protocols.^23^

Furthermore, in this study, a single dose of 30 Gy radiation was administered 2 weeks after implant installation, with the purpose of interfering with bone consolidation.^24^ This same radiation dose, based on a previous study in rabbits, demonstrated a significantly reduced volume of newly formed bone between the labels, indicating a slower rate of bone formation.^23^

The results derived from the micro-CT technique encompassed a complementary parameter used to assess the integrity and quality of the cortical bone. In this study, BV/TV exhibited a slight increase during the healing process in the Ir group; however, the observed differences were not statistically significant among the groups. It is worth noting that the scientific literature presents a divergence of results regarding this parameter, with studies that, despite revealing a subtle decrease in the Ir group, do not confirm the statistical significance in this context.^13^ On the other hand, there are research studies reporting reduced BV/TV values in irradiated groups, which exhibit statistically significant differences.^23,25,26^ Thus, the apparent disparity in the results among these studies can be attributed to variables such as the different time intervals between surgical intervention and postoperative irradiation, the administered radiation dose, and the various species of animal models used.

Stability and osseointegration must be ensured to secure a successful implant procedure. It is worth noting that the increased bone-implant contact results in improved implant stability.^27^ Within the scope of our study, all implants remained clinically stable, and there was a trend toward higher BIC in the NoIr group, although this difference was not statistically significant among the groups. Notably, the BIC values obtained in both the Ir and NoIr groups appeared to be suitable for promoting bone remodeling and, consequently, achieving osseointegration compatible with clinical implant stability, even in the presence of changes in the bone microstructure.^13,27^ However, despite the promising results reported regarding osseointegration, other authors have documented a reduction in bone formation after radiation exposure.^13,25,28^ A recent study demonstrated that BIC values were reduced when implants were placed in irradiated dog mandibles.^13^

The histomorphometric results did not reveal any statistically significant differences in the BABT between the Ir and NoIr groups. However, the NoIr group exhibited a tendency toward a greater reduction in the BABT area. These results contrast with the literature, which demonstrates a greater reduction in the BABT area in irradiated bones.^29^ The absence of a statistically significant difference, despite the clearly visible trend in the data, can be explained by the low statistical power associated with the small sample size.^30^ In addition, the histological sections represent only a portion of the area of interest.^31^ Other methods, such as reverse torque, can be employed to increase the size of the analyzed area while also evaluating parameters such as BIC.^32^

Changes in the cellular pattern of the irradiated bone can be observed,^8,33^ although our study demonstrated no qualitative differences in cell deposition around the implant between the Ir and NoIr groups. Literature suggests that ionizing radiation does not significantly alter the quantity of the bone matrix formed, and a layer of osteoblasts is found proximal to the newly formed bone tissue^34^. Conversely, some studies have reported a negative effect on osteoblasts following exposure to ionizing radiation.^8,33^ These discrepancies may be attributed to the limited sample size, which constrains a more comprehensive comparison.

In terms of the limitations of this study, it is important to note that the implants were placed exclusively in the cortical bone without the application of any load, which could potentially affect the results if such loading was present. Additionally, using a single dose of ionizing radiation in an animal research model may be considered a limitation since the healing process may be affected differently when the dose is fractionated in response to a systemic reaction. Consequently, the findings of this study cannot be directly extrapolated into clinical practice. However, our findings suggest a potential correlation between radiation responses observed in humans undergoing radiotherapy for head and neck cancers. Therefore, monitoring previously placed implants should be carefully considered in patients undergoing radiotherapy.

## Conclusion

Within the limitations of this in vitro study that tested ionizing radiation on pre-installed implants, the following conclusions were drawn:

Irradiation did not alter the bone-implant contact or bone tissue area between the implant threads.

Irradiation did not affect the bone volume around the implant when bone volume was analyzed relative to tissue volume.
